# Retraction: Herndon J.M. Evidence of Coal-Fly-Ash Toxic Chemical Geoengineering in the Troposphere: Consequences for Public Health. *Int. J. Environ. Res. Public Health* 2015, *12*, 9375–9390

**DOI:** 10.3390/ijerph120910941

**Published:** 2015-09-02

**Authors:** Paul B. Tchounwou

**Affiliations:** Molecular Toxicology Research Laboratory, Jackson State University, 1400 Lynch Street, Box 18750, Jackson, MI 39217, USA; E-Mail: paul.b.tchounwou@jsums.edu

It was brought to my attention that there are problems related to the recently published article “Evidence of Coal-Fly-Ash Toxic Chemical Geoengineering in the Troposphere: Consequences for Public Health” [1].

Together with the Chief Scientific Officer, Dr. Franck Vazquez, and the Editorial office, we re-evaluated the paper, re-assessed the comments made by the three reviewers and note the following crucial concerns:

- The value for average leachate concentration of Aluminum mentioned in Table 1 and used by the author to normalize the data presented in Figures 2, 3, 4 and 5 is incorrect is incorrect. The author uses 70,000 µg/kg, while the correct value resulting from the un-leached European coal fly ash samples measurements published by Moreno *et al*. [2]) is 140,000,000 µg/kg. This error invalidates the conclusions of the article.
- The chemical compositions obtained for rainwater and HEPA air filter dust are only compared to chemical compositions obtained for coal-fly-ash leaching experiments [2]. The author did not attempt to compare his results to chemical compositions of other potential sources. Thus, at this stage, the work is preliminary since it is not clear what the source of these chemicals is.
- The language of the paper is often not sufficiently scientifically objective for a research article.

Consequently, we have decided to retract the article. This paper is thus declared retracted and shall be marked accordingly for the scientific record.

MDPI takes the responsibility to enforce strict ethical policies and standards very seriously. We aim to ensure the publication of only truly original and scientific works. MDPI would like to apologize to the readers of IJERPH that this article was published with the errors mentioned above. We sincerely appreciate the efforts of those who bring aspects of scientific error to our attention in an effort to maintain scientific integrity.
